# Phylogeny, Structure, Functions, and Role of AIRE in the Formation of T-Cell Subsets

**DOI:** 10.3390/cells11020194

**Published:** 2022-01-07

**Authors:** Daniil Shevyrev, Valeriy Tereshchenko, Vladimir Kozlov, Sergey Sennikov

**Affiliations:** Research Institute for Fundamental and Clinical Immunology (RIFCI), 630099 Novosibirsk, Russia; tervp91@gmail.com (V.T.); vakoz40@yandex.ru (V.K.); sennikov_sv@mail.ru (S.S.)

**Keywords:** AIRE, promiscuous gene expression, tissue-specific antigens, thymic epithelial cells, variable lymphocyte receptors, T-cell receptors, B-cell receptors, evolution of adaptive immunity

## Abstract

It is well known that the most important feature of adaptive immunity is the specificity that provides highly precise recognition of the self, altered-self, and non-self. Due to the high specificity of antigen recognition, the adaptive immune system participates in the maintenance of genetic homeostasis, supports multicellularity, and protects an organism from different pathogens at a qualitatively different level than innate immunity. This seemingly simple property is based on millions of years of evolution that led to the formation of diversification mechanisms of antigen-recognizing receptors and later to the emergence of a system of presentation of the self and non-self antigens. The latter could have a crucial significance because the presentation of nearly complete diversity of auto-antigens in the thymus allows for the “calibration” of the forming repertoires of T-cells for the recognition of self, altered-self, and non-self antigens that are presented on the periphery. The central role in this process belongs to promiscuous gene expression by the thymic epithelial cells that express nearly the whole spectrum of proteins encoded in the genome, meanwhile maintaining their cellular identity. This complex mechanism requires strict control that is executed by several transcription factors. One of the most important of them is AIRE. This noncanonical transcription factor not only regulates the processes of differentiation and expression of peripheral tissue-specific antigens in the thymic medullar epithelial cells but also controls intercellular interactions in the thymus. Besides, it participates in an increase in the diversity and transfer of presented antigens and thus influences the formation of repertoires of maturing thymocytes. Due to these complex effects, AIRE is also called a transcriptional regulator. In this review, we briefly described the history of AIRE discovery, its structure, functions, and role in the formation of antigen-recognizing receptor repertoires, along with other transcription factors. We focused on the phylogenetic prerequisites for the development of modern adaptive immunity and emphasized the importance of the antigen presentation system.

## 1. Introduction

It is known that there are self-antigens with specific tissue-limited expression called tissue-specific antigens (TSAs) [1,2]. In the 1980s, it was discovered that human and animal thymus expressed various TSAs, for example, neurohypophysial hormones, some growth factors, and other antigens, including those that are expressed in the immune-privileged sites [3,4,5]. At the same time, the expression of TSA genes in the thymus is heterogenic and occurs randomly within the antigen-representing thymic cells, which provided the basis to call this phenomenon “promiscuous gene expression” (PGE) [6]. In 1997, a correlation was revealed between the level of expression of insulin in the thymus and the negative selection of self-reactive insulin-specific clones of T-cells [7,8]. Similar results were obtained in analogous studies for other antigens [9,10]. At the same time, an association was established between the autoimmune syndrome APECED (autoimmune polyendocrinopathy candidiasis ectodermal dystrophy) and mutations in the locus 22.3 on the long arm of the 21st chromosome [11,12]. Genetic studies allowed the researchers to identify a new gene located in this locus, determine its nucleotide sequence, and suggest its involvement in the regulation of the transcription because of two zinc fingers in its structure [12,13,14]. Later, it was called autoimmune regulator (*Aire*) because of a direct association between mutations in this gene and APECED syndrome [15,16]. However, only in 2001 did the analysis of a major set of TSA genes in various populations of the thymic cells show that all were expressed primarily in the mTECs population (medullary thymic epithelial cells), and that the level of their expression was closely associated with the expression of AIRE [17]. Thus, it was suggested on the role of the *Aire* gene in the presentation of self-antigens in the thymus and possible maintenance of self-tolerance.

## 2. Evolutionary Prerequisites

Phylogenetic studies showed that the appearance of the *Aire* in the evolution was associated with the development of adaptive immunity [18,19]. The emergence of vertebrates, and later, gnathostomes was closely related to two consecutive whole genomic duplications that occurred around 650–500 million years ago [20,21]. This evolutionary leap created a background for the formation of the adaptive immunity that was associated with the appearance of MHC (major histocompatibility complex) genes [22], molecular domestication of an ancient RAG (recombination-activating gene) transposon [23,24,25], and emergence of immune- and thymoproteasomes [26,27] that resulted in the development of mechanisms of specific recognition and presentation of antigens [28,29].

**Figure 1:**
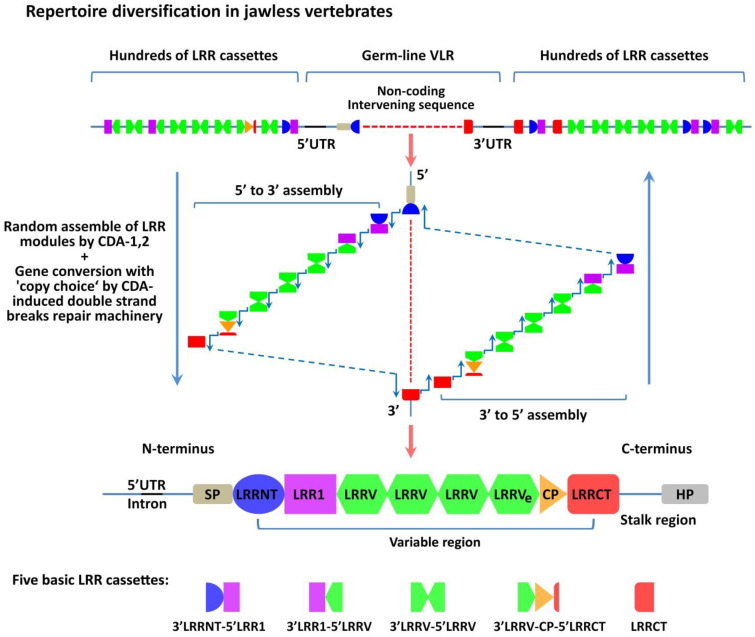
Diversification mechanism of variable lymphocyte receptors in jawless vertebrates. The germ-line VLR gene is incomplete and includes a non-coding intervening sequence flanked by hundreds of partial LRR gene cassettes. So, there are no direct coding sequences for the LRRNT, LRR1, LRRVs, LRRVe, and LLRCT modules in the germ-line configuration of the VLR genes. The assembling of functional VLR occurs during lymphocytes maturation in the thymoid by random and sequential copying of sequences from the flanking LRR cassettes into the non-coding intervening sequence in the direction from the 5’ LRRNT or from the 3’ LRRCT end. Diversification of the VLR repertoire mediates by CDA action and occurs because of the gene conversion-like process with nonreciprocal insertion of LRR cassettes into the germ-line VLR gene, and the “copy choice” mechanism, which includes an original template switch to a donor sequence of another LRR module during replication. This process is presumed to be directed by the short homologous stretches of sequence between the original and donor LRR sequences. The general array of LRR cassettes can be classified into five main structural groups, shown at the bottom of the figure. The mature VLR gene consists of several coding sequences: SP, LRRNT, LRR1, two to eight modules LRRV, LRRVe, CP, LRRCT, and the stalk region with an HP end, which anchors the VLR protein in the lymphocyte membrane. VLR, variable lymphocyte receptor; SP, signal peptide; LRR, leucine-rich repeat; LRRNT, N-terminal LRR-capping module; LRR1, first LRR; LRRV, variable LRR; LRRVe, end variable LRR; CP, connecting peptide; LRRCT, C-terminal LRR-capping module; HP, hydrophobic peptide; UTR, untranslated region; CDA, Cytidine deaminase. The illustration is not drawn to scale.

Already at the stage of jawless vertebrates (lamprey and hagfish are the only modern representatives), these animals developed thymoids, i.e., lymphoepithelial structures similar to the thymus of the higher chordates in the area of gill filaments [30]. It was established that these structures were involved in the diversification of the repertoire of antigen receptors that are called variable lymphocyte receptors (VLRs) [30]. They can directly recognize conformational antigen epitopes, but their structure is completely different from T- and B-cell receptors (TCRs and BCRs) of jaw vertebrates [31]. These receptors contain Leucine-rich repeats (LRRs) and are formed due to genomic rearrangements under the influence of the CDA1 and CDA2 (Cytidine deaminases family APOBEC)—in T- and B-like cells, respectively (Figure 1) [32,33]. It was shown that the diversification of VLRs repertoire occurs due to a random combination of various LRR segments in the final VLR assembly [31], as well as the process of gene conversion with the “copy choice” mechanism, which includes the original template switch to a donor sequence of another LRR module using short homologies during replication [33]. Enzymes CDA1 and CDA2 are distant analogs of activation-induced deaminase (AID) [34], which provides the process of somatic hypermutations in B-lymphocytes of higher chordates [35]. It should be mentioned that the expression of the *Foxn4-like* gene (orthologous of *Foxn1* gene) is also detected in the thymoids. This gene codes the transcription factor similar to FoxN1 (Forkhead box N1), which regulates the development, differentiation, and functions of thymic epithelial cells (TECs) in jaw vertebrates [36,37].

It should be noted that VLR repertoires obtained from the thymoid and peripheral blood are different. First, the thymoids often contain non-functional assemblies of VLRs that are not detected on the periphery [38]. Second, thymoids often contain VLR assemblies with a different number (2–8) of internal variable LRR modules (LRRV), while the periphery primarily contains assemblies with four LRRV modules for VLRA^+^ and VLRC^+^ lymphocytes and two LRRV modules for VLRB^+^ lymphocytes [38]. Third, non-functional VLRC assemblies that are revealed in the thymoid have a lower degree of diversity of amino acid sequence of the LRR1 module (25 amino acid residue) in comparison with functional assemblies from the peripheral blood [38].

These data indicated that the formation of the peripheral repertoire of lymphocytes in jawless vertebrates occurs as a result of selection in the thymoid [39], wherein the selection for VLR assemblies of a certain length (number of LRRV) is performed for each population VLRA^+^, VLRB^+^, and VLRC^+^, as well as for the sequence LRR1 for VLRC^+^ cells [38]. However, precise mechanisms of such selection are not known. Jawless vertebrates have no immunoproteasomes and MHC genes. Their analogs are not revealed either, which could indicate the lack of the system of antigen presentation in this group of organisms [28,29,39]. Since the adaptive immune system has to provide self-tolerance, it can be suggested that the selection of lymphocytes in the thymoid occurs through the mechanisms of negative selection of self-reactive cells during the interaction with conformational self-antigens. However, this issue is understudied and requires additional research [38,40].

**Figure 2:**
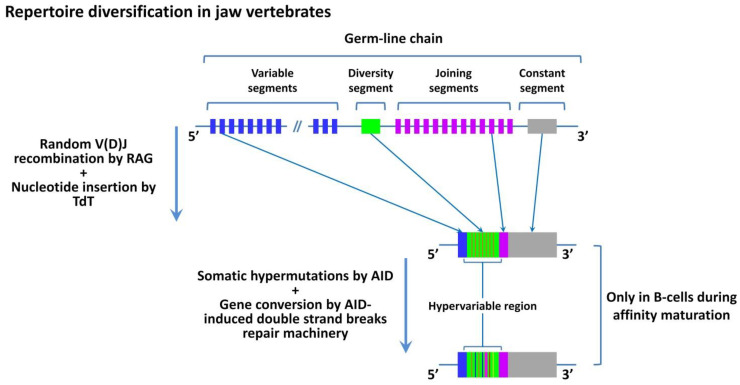
Diversification mechanism of antigen receptors (TCRs and BCRs) in jaw vertebrates. Somatic recombination is the mechanism of V(D)J segments recombination that occurs in maturating T- and B- lymphocytes at an early stage of development in the thymus and bone marrow, respectively. This process mediates by a protein complex led by the RAG protein that initiates random rearrangement of V, J, and D segments, which leads to the emergence of novel antigen-binding regions of TCRs and BCRs. An important role in repertoire diversification belongs to TdT, a template-independent DNA polymerase that randomly adds non-template nucleotides to the coding-end of an open hairpin, thereby forming an N-insert immediately after the P-insert formation. Further reorganization of the variable region is possible only in B cells during the process of somatic hypermutations that mediate by AID in the course of affinity maturation. This process relies on the gene conversion-like mechanism during the reparation of double-strand DNA breaks. RAG, recombination-activating gene; TdT, terminal deoxynucleotidyl transferase; AID, activation-induced deaminase. The illustration is not drawn to scale.

Thus, at the stage of jawless vertebrates, an adaptive immune system appears for the first time in phylogeny. It is suggested that high precision and selectivity of the immune system, which is provided due to specificity, is the most important evolutionary advantage of adaptive immunity. However, the described mechanisms of the formation of VLR repertoires in jawless vertebrates are primitive and do not provide functional possibilities of the immune system comparable to the higher chordates. Despite the potentially high diversity of VLRs that reaches 10^14^ [31], relatively high affinity and specificity of protein and glycan binding [41,42], the general VLR repertoire seems to recognize only non-self antigens. This seriously limits the ability to recognize self and altered-self antigens. This could be one of the factors that significantly reduced the evolutionary success of the majority of jawless vertebrates. Besides, the potential diversity of conformational epitopes of non-self antigens is extremely high and significantly exceeds the potential diversity of VLRs [31]. This means that there is a possibility to face a non-self antigen that will not be specifically recognized by the immune system because of the lack of the respective VLR. This can significantly reduce the effectiveness of the adaptive immune response. The described issue is solved in higher chordates due to the formation of mechanisms of antigen presentation [28,29]. The potential diversity of antigens presented by MHC is ~20^9^ [43], which is significantly lower than the potential diversity of all conformational antigens and corresponds to the observed number of unique TCRs (10^12^ and more) [44], at least in mammals. To recognize conformational antigens, higher chordates have another mechanism of specificity enhancement called affinity maturation of BCRs after a low-specific interaction with the antigen (Figure 2) [45,46]. Presently, this process or its analogs are not revealed in jawless vertebrates [29,47,48,49,50]. The emergence of the antigen-presenting system in higher chordates also solved the issue of the recognition of self/altered-self due to the mechanism of presentation of self-antigens in all the cells of an organism. The appearance of damaged or altered-self antigens in an MHC-I complex induces a specific immune response or elimination of cells that carry these antigens [51,52,53]. The appearance of this mechanism required the development of the tools of precise “calibration” of initial TCR repertoires, formed in the process of genome rearrangement. It was also associated with the emergence of mechanisms of positive and negative selection in the thymus, the appearance of T-regulatory cells (Treg), and functional division of the general population of T-cells into CD4^+^ and CD8^+^ subpopulations [28,29,54,55,56,57]. These conditions were fulfilled due to the evolution of the thymus and appearance of an Aire-led genetic system that provides the presentation of various self-antigens in the thymus, participates in the processes of selection of lymphocytes, and is involved in the formation of repertoires of Treg, CD4^+^, and CD8^+^ lymphocytes [18,58,59,60,61].

Thus, *Aire* not only provides self-tolerance but also creates the background for the enhancement of specificity of the adaptive immunity. In other words, it is one of the factors that enhances the ability of the immune system to recognize self, altered-self, and non-self to a high degree of precision. It should be mentioned that evolutionary development of the antigen-presenting system was closely associated with the appearance of *Aire* [18,28], which could reflect the functional significance of this gene in the context of the phylogenetic development of adaptive immunity. It worth to note that the *Aire* gene is also revealed in basking sharks (the oldest representatives of cartilaginous fish and jaw vertebrates) that have the main genes that provide the presentation of antigens on the periphery and in the thymus [28,29].

## 3. Structure and Localization of AIRE

As it was mentioned above, the gene AIRE is located in the locus 22.3 on the q-arm of chromosome 21 in humans [13,14]. In 1997, as a result of cloning and sequencing of the *Aire* gene, its sequence was established that included 12.9 thousand base pairs and 14 exons, and encoded mRNA 1635 nucleotides long [16]. The translation of *Aire* mRNA leads to the synthesis of AIRE protein, which is a transcription regulator that consists of 545 amino acid residues and has a weight of 56 kDa [62].

**Figure 3:**
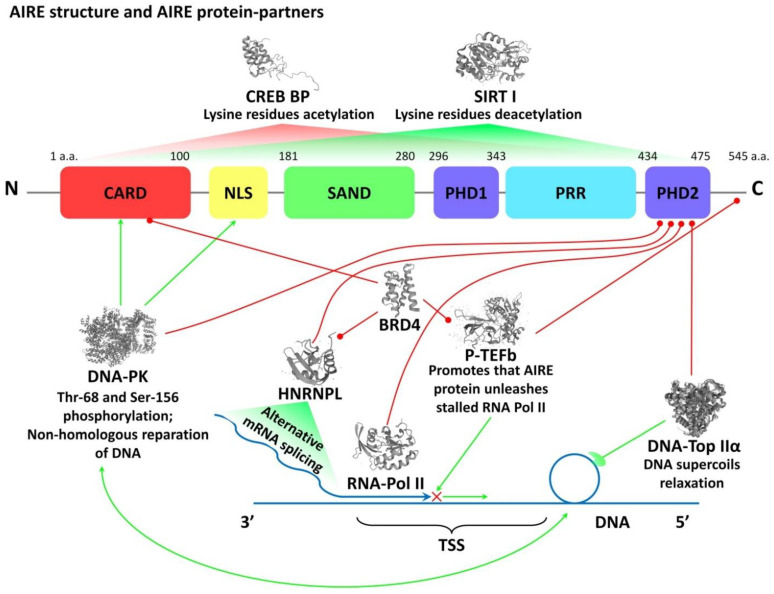
Molecular structure and functions of AIRE transcription regulator. Explanations are in the text. Red lines show spatial contacts in the AIRE multimolecular complex. Green arrows show functional interconnections in the AIRE multimolecular complex. AIRE, autoimmune regulator; CARD, caspase activation and recruitment domain; NLS, nuclear localization signal; SAND domain is named after a range of proteins: Sp100, AIRE-1, NucP41/75, DEAF-1; PHD, plant homeodomain; PRR, Proline-rich region; CREB BP, CREB binding protein; CREB, cAMP response element-binding protein; SIRT, Sirtuin; DNA-PK, DNA-dependent protein kinase; HNRNPL, heterogeneous nuclear ribonucleoprotein L; RNA-Pol II, RNA polymerase II; BRD4, Bromodomain-containing protein 4; P-TEFb, positive transcription elongation factor b; DNA-TOP IIα, Type II DNA topoisomerase; TSS, transcription start site. Three-dimensional structures of proteins were obtained from https://www.uniprot.org/ (accessed on 15 October 2021).

The transcription regulator AIRE contains several domains in its structure. It can bind with chromatin, regulates the expression of numerous genes, and exerts a pleiotropic effect. Starting with N terminus, AIRE protein contains a caspase activation and recruitment domain (CARD) that is responsible for oligomerization of a protein [63], a domain of nuclear localization signal (NLS) that mediates nuclear import and export of AIRE [64], a domain SAND (SP100, AIRE1, NucP41/P75, and DEAF1) involved in binding AIRE with DNA and interaction with other transcription factors [65], and Proline-rich sequence that does not carry specific functions and mediates the interaction with various signaling pathways. Besides, it has two domains PHD1 and PHD2 (plant-homeodomain fingers) [18] that are responsible for the interaction with histones and DNA-dependent protein kinase and involved in the activation of various genes transcription (Figure 3) [60,61,66].

A transcription regulator AIRE is localized primarily in the cell nucleus, wherein it is visualized as small aggregates in the nuclear bodies that are evenly distributed in the nucleoplasm [64]. It is also contained in the cytoplasm, wherein it forms a 3D network connected with the nuclear matrix, which allows it to affect the functional domains of chromatin and change the availability of the sites that regulate gene expression [64]. Considering the dependence of the structural organization of AIRE from the cellular cycle at the subcellular level, it can be suggested that the functional activity of AIRE is controlled by temporospatial patterns and is associated with the changes in the organization of a 3D AIRE network inside a cell [67].

The highest expression of AIRE detects in the thymus. The lower AIRE expression can be detected in other tissues—in nervous and lymphatic tissues, bone marrow, skin, etc., wherein AIRE, along with other transcription factors, provides the expression of single genes via different epigenetic mechanisms in various periods of ontogenesis [68]. However, the most complete description of its functions and mechanisms is provided for terminally differentiated mTECs^hi^ that present various TSAs to maturing lymphocytes and maintain the processes of selection [60,61,68]. Medullary TECs develop from the thymic epithelial precursor cells (TEPCs) that also give rise for cortical thymic epithelial cells (cTECs) [69]. Both cTECs and mTECs populations are involved in the maturation and selection of lymphocytes in the thymus [70]. The studies on mice showed that the differentiation of TECs began at the early stages of embryonic development and the expression of AIRE in mTECs^hi^ was detected already on day 11–14 of gestation [16,61].

**Figure 4:**
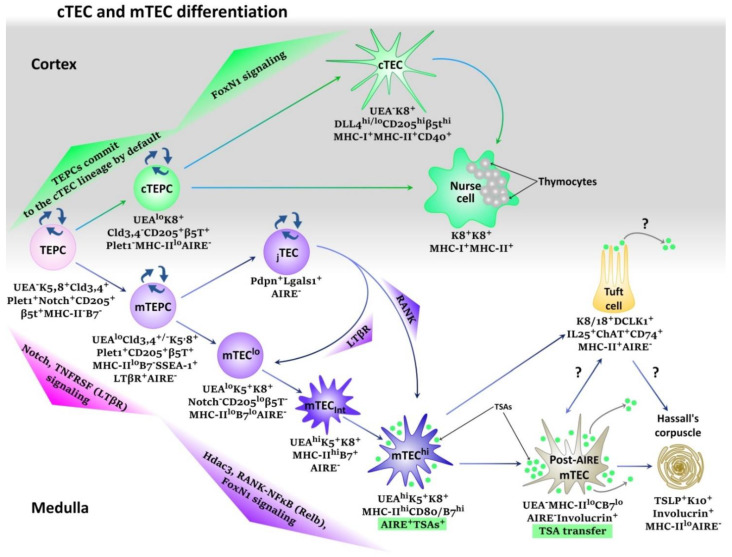
Recent technological approaches made it possible to identify a high level of TECs heterogeneity and reveal the existence of various cTEC and mTEC subpopulations with distinct molecular and functional characteristics. This figure shows a simplified scheme of cTEC/mTEC differentiation at the embryonic period. Thymic epithelial progenitor cells (TEPCs) are supposed to give rise to two main lineages of thymic epithelial cells—cortical and medullary. TEPCs colonize the thymus in early embryogenesis, have spontaneous Notch expression, and are capable of self-renewal because of a high level of Notch-signaling due to the lateral induction mechanism. TEPCs are assumed to commit to the cTEC lineage by default without a Notch signal, becoming renewable cortical progenitor cells (cTEPCs). Low Notch-signal at this stage can upregulate FoxN1 and induce differentiation of cTEPCs to cTECs or Nurse Cells. TEPCs that experience a low level of Notch-signaling commit to the mTEC lineage and form renewable medullary progenitor cells. Subsequent differentiation of mTEPCs occurs during “thymic crosstalk” between epitheliocytes and maturating lymphocytes and relies on FoxN1-signaling, TNFRSF, and RANK-NF-κB pathways. That is accompanied by upregulation of UEA, MHC-II, and B7 cell surface expression. Then Histone deacetylase 3 downregulates Notch-signaling to result in differentiation of functional AIRE^+^mTECs^hi^, which soon undergo terminal differentiation or apoptosis and form post-AIRE structures as post-AIRE cells, Tuft-cells, or Hassall’s corpuscles. These post-AIRE structures have an essential role in the TSA presentation to maturating lymphocytes, particularly TSA transfer to dendritic cells is critical for optimal presentation of some self-antigens. In addition, recent studies have shown that in the thymus, at least in mice, there are Pdpn^+^ junctional thymic epithelial cells (jTECs), which are localized at the cortico-medullary junction. These cells are renewable and give rise to mTEC^lo^ and AIRE^+^mTEC^hi^ subsets in adulthood.

However, the mechanisms of development of two main cell lineages of thymic epithelial cells (cTECs and mTECs) are presently understudied. This is a complicated process that involves various transcription factors at different stages of ontogenesis. [71]. At the early stage of embryogenesis, Notch-signaling is the most significant factor for homeostasis and TEC differentiation (Figure 4) [72,73,74]. It is suggested that Notch-mediated mechanisms of lateral inhibition and lateral induction determine the development of medullar and cortical lineages of TECs [75,76,77,78]. In the absence of Notch-signaling, the TEPCs commit towards cortical thymic epithelial precursors cells (cTEPCs) that form a self-renewable cells pool. [73,74]. Weak Notch-signaling is necessary for committing TEPCs towards the medullary thymic epithelial precursor cells (mTEPCs) that are also capable of self-renewal. [73,74]. At the same time, strong Notch-signaling leads to the arrest of TEPCs maturation, which provides homeostatic maintenance of this cell population, at least, in the embryogenesis [73,74]. It is suggested that populations with cTEPCs and mTEPCs maintain the pool of mature thymic cortical and medullar epitheliocytes [79,80]. Further differentiation of mTEPCs depends on the signaling pathways LTβR, Relb, and FoxN1-signaling, and is associated with lymphostromal interactions between maturing lymphocytes and thymic epitheliocytes. This process is also called “thymic crosstalk” [61,80,81,82,83,84,85]. The differentiation of mTECs begins after the deactivation of the Notch-signaling pathway with Hdac3 histone deacetylase that induces the RANK-NFκB-independent transcriptional program typical for maturation of AIRE^+^mTEC^hi^ cells. This is associated with the induction of the expression of MHC-II, molecules of co-stimulation, and transcription regulator AIRE, which finally initiates the presentation of TSAs [86]. The stage AIRE^+^mTEC^hi^ is short, these cells are prone to apoptosis and terminal differentiation that leads to the appearance of such populations as post-AIRE mTECs, Tuft-cells, and other understudied populations, as well as Hassall’s corpuscles [80,87,88,89]. At the same time, precise functions and associations between these post-AIRE structures are to be established.

Detailed mechanisms of differentiation of cTECs are not known. It is suggested that at a certain stage, weak Notch-signaling intensifies FoxN1-signalling in cTEPCs, which leads to the maturation of functional cTECs or Nurse-cells [71,73,74]. The latter uses a membrane to cover simultaneously several dozens of maturing thymocytes and present antigens to them as a part of MHC molecules taking part in the processes of positive and negative selection [90,91].

It should be mentioned that apart from the thymus, the expression of AIRE was detected on the periphery in some antigen-presenting cells (APCs) that were capable of presenting TSAs, although to a lower degree than mTECs. These cells were called Extrathymic AIRE-expressing cells (eTACs) [92]. The results of the studies on transgenic mice suggested the involvement of eTACs in the induction of CD4^+^ lymphocytes tolerance in the secondary lymphoid organs [92]. However, recent studies on human eTACs, performed with a transcriptome analysis at the single-cell level, showed that eTACs belonged to the population of dendritic cells (DCs) that transitory express AIRE during maturation and have potential to the stimulation of T-lymphocytes [93]. At the same time, unlike the thymus, the expression of AIRE in human eTACs was not associated with PGE of TSAs [93]. According to some authors, AIRE can participate in the induction of peripheral tolerance via the control of TLRs expression and influence the maturation of APCs [94]. In general, recent studies showed that on the periphery, AIRE acted as a coordinator in various transcriptional programs. This is supported by its intracellular spatial organization and access to various parts of the genome [67,94,95]. Besides, it was shown that in the embryonic period, AIRE participated in the assembly of the spindle apparatus and was an important factor of a mitotic cycle [96]. Still, a recent study on mice models showed that selective deletion of eTACs (but not mTECs) in mothers led to an immune-mediated disorder during the intrauterine development of the fetus in mice with both allogeneic and syngeneic pregnancy [97]. In addition, a recent study revealed a population of tolerogenic eTACs that have broad gene expression with a range of TSAs and high homology to mTECs. These cells can potentially influence self-representation and tolerance at the periphery [98]. Thus, the results obtained during the past decade are controversial and do not allow the researchers to exclude the possibility of the existence of the mechanism of induction of AIRE-mediated peripheral tolerance. Probably, the level of AIRE expression affects what functions it will execute in a cell. It is well-known that mTECs have a high expression of AIRE, while the peripheral tissues and eTACs have a significantly lower expression of AIRE [68]. At the same time, it can be stated that AIRE is a coordinator of transcription, and it participates in the differentiation of various tissues at different stages of ontogenesis.

## 4. Molecular Mechanisms of AIRE Action

It is known that AIRE differs from canonical transcription factors and acts as a part of the multi-molecular complex providing stochastic, and at the same time, coordinated expression of TSAs in mTECs (PGE) [99]. The expression of a certain TSA in a certain period is detected only in 1–3% of mTECs and has a stochastic nature. At the same time, each mTEC can express various TSAs simultaneously [6]. Along with this, there is a possibility of co-expression of a certain set of TSA genes in one mTEC, which is associated with the capability of AIRE to induce the expression of TSA genes located in one chromosomal area or to form the networks between chromosomes through synchronization of gene expression from certain loci of various chromosomes [100,101]. A spatial network structure of AIRE, associated with the nuclear matrix, significantly contributes to these processes [67].

A multi-molecular complex includes several AIRE “protein-partners” (Figure 3) [60,61]. The first identified protein-partner was CREB-binding protein (CBP) that stabilizes the network structure of AIRE due to acetylation of lysine residues and suppresses its functional activity [102,103]. Deacetylation of lysine residues with deacetylase Sirtuin 1 (SIRT I) leads to the activation of AIRE [104]. Another AIRE partner is a DNA-dependent protein kinase (DNA-PK) that phosphorylates residues of threonine and serine (in sites 68 and 156, respectively). As a part of a multi-molecular complex, it is involved in a non-homologous reduction of DNA double-strand breaks [105,106]. Such temporary breaks occur under the influence of another AIRE partner DNA-Top IIα, which relaxes supercoils in the transcription start sites (TSSs) of TSAs and simplifies the synthesis of their mRNA [61,107]. Another important AIRE partner is a Positive transcription elongation factor b (P-TEFb), which releases RNA-Pol II (stalled in the proximal areas of the promoter) and provides productive elongation and splicing of mature mRNA [108]. In this context, it should be mentioned that in the majority of human genes, right after the initiation of transcription, RNA-Pol II gets temporarily stalled under the influence of certain factors. To continue the synthesis of mRNA, P-TEFb should be involved [109]. It was established that AIRE recruits P-TEFb in the TSSs of the target genes and activates the synthesis of TSAs mRNA [61,107]. Besides, another AIRE partner is a heterogeneous nuclear ribonucleoprotein L (HNRNPL). It is involved in the elongation and alternative splicing of mRNA and suggests AIRE involvement in the diversification of mRNA [110]. An important role in the maintenance of the structure of a macromolecular complex is played by a domain Bromodomain-containing protein 4 (BRD4), which directly connects AIRE with P-TEFb and HNRNPL and forms a complex for the release of the stalled RNA-Pol II [111]. In addition, it was shown AIRE, as a part of the multimolecular complex, can interact and activate different transcriptional regulators localized on super-enhancers through DNA-Topoisomerase I [112]. A recent study performed with genome-wide, high-resolution chromosome-conformation capture experiments confirmed that AIRE affects chromatin organization through the widespread promotion of super-enhancer–promoter loops. AIRE can promote chromatin loops extrusion in super-enhancer regions through association with the cohesin loader and activation of cohesin’s enzymatic subunits. At this time, the architectural chromatin protein (CTCF) relatively depletes, which maintains the super-enhancers loops extrusion process [113]. Thus, recruiting a multi-molecular AIRE complex to the transcription start sites leads to the activation of PGE in the majority of genes in mTECs.

## 5. The Role of AIRE in the Formation of the TEC Transcriptome and Immunopeptidome 

**Figure 5:**
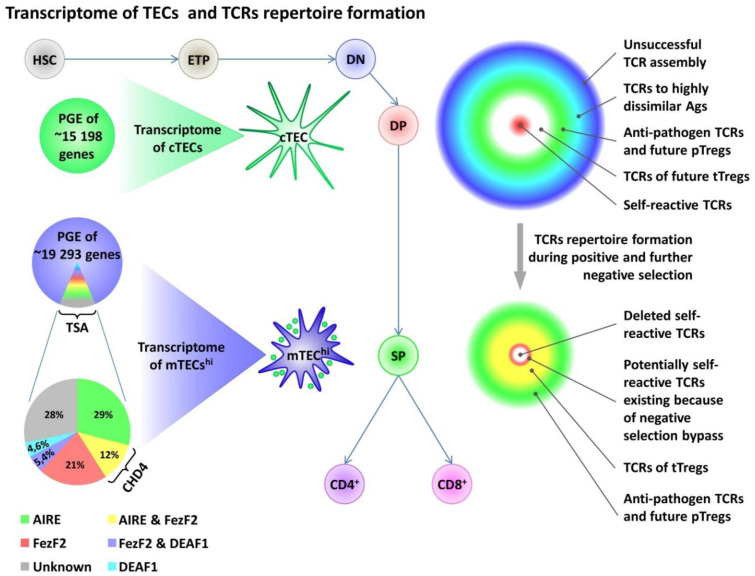
TECs transcriptome and TCRs repertoire formation in the thymus. PGE is common to cortical and medullary TECs. However, mTECs have higher PGE than cTECs due to the expression of several transcriptional factors as AIRE, FezF2, and DEAF1, which also provide transcription of thousands of TSAs genes in mTECs. The contribution of these transcription factors to TSAs expression is shown in the Figure as a percent of total TSAs expression in mTEC^hi^ population. On the left, changes in the TCR repertoire during positive and negative selection are shown. PGE, Promiscuous Gene Expression; cTECs, cortical thymic epithelial cells; mTECs, medullary thymic epithelial cells; AIRE, Autoimmune Regulator; FezF2, Forebrain Embryonic Zinc Finger-Like Protein 2; DEAF1, Deformed Epidermal Autoregulatory Factor 1; HSC, Hematopoietic stem cells; ETP, Early T-cell Precursor; DN, double-negative stage, DP, double-positive stage, SP, single-positive stage of thymocytes maturation.

The genomes of a human and a mouse contain approximately 24,000 genes; ~19,293 of them are expressed in the pool of mature mTECs^hi^, which is more than 87% of all genes that encode proteins. There are no cells that have such transcriptional diversity [60,114,115,116]. At the same time, in the pools of immature mTECs^lo^ and post-AIRE mTECs, a significantly lower number of genes are transcribed (on average, 16,951 genes, which is 76% of all protein-coding genes), and in the population of cTECs, this number is around 15,198 genes (68% of all protein-coding genes) [114,115,117]. To compare, in other tissues, around 7500 genes are transcribed [118]. Thus, both medullar and cortical epithelial cells of the thymus have PGE. However, the highest expression of the genes is observed in the population of mTECs^hi^ due to the transcription regulator AIRE that additionally induces the expression of approximately 10–20% of genes [114,115]. This is possible because AIRE has 42,124 sites of binding throughout the genome, including super-enhancers, wherein it interacts with histones and opens chromatin in the composition of a multi-molecular complex. This allows AIRE to induce simultaneously the expression of numerous genes [112,113]. The analysis at the single-cell level showed that a separate cell mTEC^hi^ could express more than 5262 genes simultaneously. Some of them encode TSAs [113]; at the same time, mTEC^hi^ express various sets of TSA genes, which underlies PGE [6,114,115]. Besides, AIRE is involved in the control of differentiation programs and suppresses around 66 genes in mTECs and ~309 genes in cTECs not affecting the expression of housekeeping genes [116,119]. In total, AIRE induces the expression of ~3980 genes in mTECs^hi^; among them, ~594 are completely AIRE-dependent. The expression of the rest ~3386 genes is AIRE-enhanced under the influence of a multi-molecular complex. The transcription of the rest genes is AIRE-independent [114,115]. At the same time, only 25% of AIRE-induced genes encode TSAs, which is around 40% of all TSA genes that are transcribed in the population mTECs^hi^ (Figure 5) [114,115].

For effective elimination of potentially self-reactive T-cells in the thymus, the expression of the maximal number of self-antigens is required at the stage of negative selection. As it was noted above, PGE in mTECs^hi^ provides a transcription of the majority of protein-encoding genes. However, a total diversity of proteins in a human organism several-fold exceeds the total number of genes [120]. Thus, additional mechanisms are needed to increase the diversity of the presented genes in the thymus. One of these mechanisms is associated with a significant AIRE-induced enhancement of alternative splicing in the population of mTECs^hi^ [121,122], as well as some AIRE-independent ways that play an important role in the maintenance of splicing complexity in AIRE^-^ populations of TECs [114]. Nevertheless, alternative splicing determines only around 40% of protein modifications. The rest modifications of proteins in the proteome depend on single amino acid polymorphism (SAP) and post-translational modifications (PTM) [120]. It was established that the thymus nearly completely lacked TSAs that underwent post-translational modifications, which explained a lower level of induction of central tolerance to such antigens. It is also reflected in the pathogenesis of some autoimmune diseases associated with the formation of secondary modified proteins [123,124,125].

Autophagy also plays an important role in the formation of immunopeptidome mTECs [126,127,128]. It contributes to the presentation of endogenous antigens to CD4^+^-lymphocytes due to the mechanism of non-classic cross-presentation, when the self-genes synthesized during PGE get inserted in MHC-II molecules [126,129]. It was shown that disturbances in the process of autophagy led to a decrease in the effectiveness of negative selection [126,128]. Probably, high expression of AIRE in mTECs^hi^ can enhance the process of autophagy [130]. Another AIRE-dependent mechanism that affects the effectiveness of negative selection is associated with a transfer of self-antigens with exosomes from mTECs^hi^ and post-AIRE structures to dendritic cells (DCs) of the thymus, and for some antigens, this pathway of presentation is the main one [131,132]. Thus, the transfer of antigens increases the availability of numerous TSAs for the pool of maturating thymocytes and contributes to the processes of selection [131,133].

It was mentioned earlier that AIRE-independent mechanisms provide the presentation of the majority of antigens to the maturating thymocytes. Forebrain embryonic zinc finger-like protein 2 (FezF2) is another transcription factor that directly induces the expression of AIRE-independent TSA genes in mTECs [134,135]. A defect or lack of FezF2 in mTECs in mice leads to severe autoimmune manifestations that are different from those that are observed in mice with AIRE deficits. This is explained only by a partial overlap of TSA spectra induced by these factors [70,134]. Transcription factor FezF2 contains six zinc-finger domains at the C-terminus and an Engrailed Homology-1 domain at the N-terminus, which interacts with open chromatin and directly recognizes specific DNA areas [70,134]. It was established that FezF2 controlled the expression of around 21% of TSA genes in mTECs^hi^, and ~12% of TSAs were controlled by FezF2 together with AIRE in cooperation with helicase CHD4 (Figure 5) [134,136]. At the same time, a defect in CHD4 also leads to the development of autoimmune manifestations, malformation of Treg lymphocytes, and changes in the BCR repertoire [136]. Thus, in total, AIRE and FezF2 control the expression of around 60% of TSA genes [134,137], which suggests the presence of additional mechanisms or transcription factors that provide the expression of TSAs in the thymus. Thus, a recent study showed that the expression of around 4.6% of TSA genes was controlled by a transcription factor DEAF1 (deformed autoregulatory factor 1) that was revealed in all APC populations of the thymus with the highest level of expression in CD141^+^DCs [135]. The mechanisms of presentation of other TSAs are understudied and could be associated with a transfer of some antigens to the thymus from the periphery with DCs. It was shown that Sirpα^+^DCs were localized in the corticomedullary zone and present antigens captured from the blood flow or acquired in the peripheral tissues before migration to the thymus [138]. Another DC population is represented by B220^+^pDCs that present antigens obtained primarily on the periphery before migration to the thymus [138,139,140]. Thus, the formation of the transcriptome and immunopeptidome in the thymus is controlled by several transcription factors and associated with the mechanisms of alternative splicing of mRNA TSAs, as well as a transfer of antigens from the periphery by various DC populations [141]. As a result, a unique landscape of antigen presentation is formed, which provides a positive and negative selection, and is involved in the formation of TCR repertoires of T-cells (Figure 5).

## 6. The Role of AIRE in the Formation of TCR and BCR Repertoires

As it was noted above, the main role in the formation of an immunopeptidome in the thymus is played by PGE in TECs. This is a complicated and understudied process and AIRE plays a key role in it [60,114,115,116,117]. Due to this process, the maturing thymocytes meet to the majority of antigens and go through the positive and negative selection [142]. As a result, various TCR repertoires of CD4^+^ and CD8^+^ cells are formed that target the recognition of non-self antigens and can recognize self/altered-self with a high degree of precision. Besides, they provide self-tolerance due to a population of Treg cells with TCR repertoire that recognizes self-antigens [143,144]. In other words, the landscape of antigen presentation in the thymus plays a key role in the formation of landscapes for the recognition of T-cells and provides the “calibration” of TCR repertoires. It is necessary so that the adaptive immune system could recognize self/altered-self and non-self, effectively eliminate non-self and altered-self antigens, and maintain self-tolerance [143,145]. It could be a significant advantage of the adaptive immunity of higher chordates in comparison with jawless vertebrates. At the same time, the adaptive immune system of higher chordates has its limitations. Thus, in the process of positive selection, only those lymphocytes survive that are capable of recognizing self-antigens in the MHC molecules on the cTEC surface [55]. As a result, the final TCR repertoire contains a limited number of TCRs with high specificity to non-self antigens that are highly dissimilar from self-antigens [55]. Even though the highest immunogenicity is observed in peptides different from the self [146,147], the immune system is not capable of recognizing a significant share of peptides revealed in various pathogens [55]. It was established that rare peptides or peptides that are not met in the human proteome were poorly recognized by the immune system, and the repertoire of naive T-cells lacked TCRs specific to such antigens [55]. It is interesting that such gaps in the TCR repertoires do not overlap due to cross-reactivity and can create the background to a decreased immune response to various infectious agents [55]. This seems especially important in the context of homeostatic proliferation that significantly affects TCR repertoires contributing to the expansion of those T-cell clones that have the highest affinity to self-antigens [148,149,150]. It is suggested that this process leads not only to an increase in the self-reactive clones on the periphery but also to a decrease in the number of clones capable of recognizing non-self antigens that significantly differ from self-antigens from the self-proteome. Homeostatic proliferation could underlie age-related changes that are associated with a decrease in the general reactivity of the immune system and an increased risk of oncological diseases [151,152,153,154].

It is well-known that negative selection is critical for self-tolerance [155]. This process results in the elimination of T-cells with relatively high affinity to self-antigens, and cells with moderate affinity form a pool of Treg lymphocytes. This occurs due to the AIRE-dependent presentation of TSAs to the maturating thymocytes in the medullary area of the thymus. It not only provides transcription and alternative mRNA splicing of AIRE-dependent TSAs but also coordinates the intercellular interactions, manages cell migrations, takes part in TSA transfer, and controls the programs of TEC differentiation [60,61,142]. As it was mentioned above, AIRE primarily induces the expression of TSAs of organs and tissues that get affected in patients with APECED syndrome (pancreas, adrenal glands, stomach, liver, etc.) [114,115]. The expression of the rest TSAs is induced by AIRE-independent mechanisms. Since AIRE provides the presentation of 20% to 40% of TSAs in the thymus [114,115,134], it can be suggested that the Treg pool of the thymic origins contains the same percent of TCRs specific to AIRE-dependent antigens. It should be noted that due to the formation of the pool of Treg lymphocytes, AIRE indirectly affects the formation of the peripheral repertoire of BCRs [156,157]. It was shown that AIRE deficit led to a condition when the pool of effector CD4^+^ lymphocytes accumulated numerous TCRs that normally belong to the Treg pool [156,158]. It was established that in patients with APECED syndrome or AIRE-deficient mice, the negative selection of self-reactive B-cells was impaired on the periphery, which is normally provided by an inhibiting influence of Treg lymphocytes [156,159]. These patients accumulate mature naïve self-reactive B-cells that produce a wide range of autoantibodies with low specificity to various AIRE-dependent antigens, which is manifested in the clinical picture of patients with APECED syndrome [156,157,159].

It should be noted that AIRE-dependent mechanisms of TSA presentation are not completely self-sufficient and not functionally redundant. There are more AIRE-independent TSAs and they are presented in various organs and tissues. Thus, disorders in AIRE-independent PGE should also lead to autoimmunity because of improper negative selection and formation of a defective repertoire of Treg lymphocytes, as well as change the BCR repertoire [114,134,135,136].

Thus, PGE plays a leading role in the formation of immunopeptidome of the thymus and is maintained by AIRE-dependent and AIRE-independent mechanisms. However, the control of intercellular interactions, management of thymocytes migration, maintenance of antigen transfer, and increase in the diversity of the presented antigens are AIRE-dependent and affect the final landscape of antigen presentation. In turn, it forms TCR repertoires of CD4^+^, CD8^+^, and Treg lymphocytes, and indirectly affects the peripheral BCR repertoire of B-cells.

## 7. Conclusions

The history of the development of adaptive immunity dates back more than 650 million years and is associated with two consecutive whole-genome duplications. It provided the background for the development of the system of repertoire diversification of antigen-recognizing receptor and the formation of the mechanisms of antigen presentation. Jawless vertebrates were the first to develop the system of specific recognition of the majority of potential antigens. It was based on the excessive clonal diversity of lymphocytes that was achieved due to controlled mutagenesis of antigen receptors. However, a lack of mechanisms of antigen presentation in jawless vertebrates imposed limitations on the capability of the immune system to recognize self-altered or damaged self-antigens that can appear as a result of mutations or infections. It could have significantly reduced the evolutionary potential of this group of organisms. Presently, only representatives of the cyclostome superclass have this type of immune system. The next evolutionary leap in the development of immunity was associated with the appearance of the mechanisms of antigen presentation. Already in the early Paleozoic period, around 525 million years ago, cartilaginous fish appeared that had all advanced features of modern adaptive immunity. Basking sharks, ancient representatives of this class of animals that survived to the present days, have a thymus, spleen, fully formed repertoires of T- and B-cells, mechanisms of affinity maturation of antibodies, and a developed system of presentation of self and non-self antigens in the MHC-I and MHC-II complexes due to the presence of immune- and thymoproteasomes. This became possible not only due to the appearance of the diversification mechanisms of antigen-recognizing receptor repertoires but also as a result of the development of the antigen presentation system that is closely associated with AIRE functions and mechanisms of PGE in the thymus. The latter creates conditions for precise “set up” of antigen-recognizing receptor repertoires and allows the immune system to recognize self, altered-self, and non-self with high precision and to maintain self-tolerance. It is suggested that it improves the capability of the immune system to provide genetic homeostasis and maintain multicellularity, which provides certain evolutionary advantages to animals with such type of immunity. It is confirmed by the fact that the basics of the immune system functioning formed 500 million ago were fixed in the course of evolution. There were no significant aromorphoses observed that could have altered the principles of the immunity performance.

However, the modern adaptive system has certain drawbacks. For example, the mechanisms of positive selection impose significant limitations on the forming TCR repertoire depleting it with lymphocyte clones that are not capable of recognizing antigens that significantly differ from the self-antigens of the organism proteome. This limitation can be manifested as a decrease in the effectiveness of the immune response to certain pathogens. At the same time, the forming repertoire of thymocytes preserves numerous potentially self-reactive T-cells that have to be eliminated at the stage of negative selection to maintain self-tolerance. The effectiveness of this process depends on the diversity of the presented self-antigens that is achieved due to AIRE-dependent and AIRE-independent mechanisms of PGE. This is primarily a random process that carries certain risks for cells and requires strict coordination at the intracellular and intercellular levels for the maintenance of optimal conditions of negative selection. The role of such a coordinator is played by the AIRE-led system of transcription factors that appears in the course of evolution parallel to the development of mechanisms of antigen presentations. This could reflect the functional meaning of the *Aire* gene in the context of phylogenetic development of adaptive immunity.

In general, AIRE can be considered as a transcriptional super controller that provides the expression, presentation, and transfer of numerous TSAs, regulates intercellular interactions and “thymic crosstalk”, and takes part in the diversification of the thymus immunopeptidome due to an enhancement of alternative splicing. Thus, it contributes to the formation of repertoires of antigen-recognizing receptors of CD4^+^, CD8^+^, Tregs, and B-cells.

## Data Availability

I would exclude this statement since this is a review article.
